# Acknowledgement to Reviewers of *Toxics* in 2015

**DOI:** 10.3390/toxics4010002

**Published:** 2016-01-21

**Authors:** Toxics Editorial Office

**Affiliations:** MDPI AG, Klybeckstrasse 64, CH-4057 Basel, Switzerland; toxics@mdpi.com

The editors of *Toxics* would like to express their sincere gratitude to the following reviewers for assessing manuscripts in 2015. 

We greatly appreciate the contribution of expert reviewers, which is crucial to the journal’s editorial decision-making process. Several steps have been taken in 2015 to thank and acknowledge reviewers. Good, timely reviews are rewarded with a discount off their next MDPI publication. By creating an account on the submission system, reviewers can access details of their past reviews, see the comments of other reviewers, and download a letter of acknowledgement for their records. Feedback from reviewers shows that most see their task as a voluntary and mostly unseen work in service to the scientific community. We are grateful to our reviewers for the contribution they make.

Albers, James W.
Ale, Albert
Arcury, Thomas
Badot, P.M.
Balayssac, David
Batteux, Frédéric
Bouris, Dimitris
Campbell, Kathleen C.M.
Carta, Manolo
Chen, Jiangang
Chiurchiù, Valerio
Chobot, Vladimir
Coloso, Claudio
Cory-Slechta, Deborah A.
Cuesta, Alberto
Deurwaerdère, Philippe
Escher, Beate I.
Fabiani, Roberto
Freeman, Jennifer L.
Fujita, Masayuki
Griffith, Darren M.
Grisold, Wolfgang
Hadjigeorgiou, Georgios
Hanson, Mark L.
Himeno, Seichiro
Hoyer, Friedrich Felix
Huber, Heinrich J.
Kanda, Isao
Kerchev, Pavel
Kim, Seok Jin
Kim, Jeong-Han
Kiyama, Ryoiti
Kojima, Hiroyuki
Krishnan, Arun
Laforenza, Umberto
Lanvers-Kaminsky, Claudia
Lin, Zhoumeng
Mannelli, Lorenzo Di Cesare
Martin, Mary Beth
Mayer, Gregory D.
Mograbi, Baharia
Moretto, Angelo
Morfini, Gerardo
Muggia, Franco
Myridakis, Antonis
Panoff, Jean-Michel
Park, Susanna B.
Dougherty, Patrick M.
Paulsen, P.
Posthuma, Leo
Prado, Rafael de
Price, Timothy Jay
Prozialeck, Walter C.
Puddu, Paolo Emilio
Robert, Jacques
Rogers, John M.
Ross, M.K.
Ruiz, José
Sandalio, Luisa M.
Sanz-Medel, Alfredo
Shafer, Timothy J.
Silva, Manori J.
So, Hong-Seob
Staff, Nathan P.
Talbott, Evelyn O.
Thewke, Douglas P.
Trevisan, Andrea
Tsuboi, Yoshio
Ugedo, L.
Vallero, Daniel A.
van Thriel, Christoph
Vilholm, Ole Jakob
Wallace, David
Waring, Rosemary
Wessolek, Gerd.
Wolff, Mary
Zoroddu, Maria Antonietta

